# Tipping point realized in cod fishery

**DOI:** 10.1038/s41598-021-93843-z

**Published:** 2021-07-12

**Authors:** Christian Möllmann, Xochitl Cormon, Steffen Funk, Saskia A. Otto, Jörn O. Schmidt, Heike Schwermer, Camilla Sguotti, Rudi Voss, Martin Quaas

**Affiliations:** 1 Institute of Marine Ecosystem and Fisheries Science (IMF), Center for Earth System Research and Sustainability (CEN), Hamburg University, Hamburg, Germany; 2 Center for Ocean and Society (CeOS), Christian-Albrechts-University Kiel, Kiel, Germany; 3 International Council for the Exploration of the Sea (ICES), Copenhagen, Denmark; 4 German Centre for Integrative Biodiversity Research (iDiv), Halle-Jena-Leipzig, Leipzig, Germany

**Keywords:** Fisheries, Population dynamics

## Abstract

Understanding tipping point dynamics in harvested ecosystems is of crucial importance for sustainable resource management because ignoring their existence imperils social-ecological systems that depend on them. Fisheries collapses provide the best known examples for realizing tipping points with catastrophic ecological, economic and social consequences. However, present-day fisheries management systems still largely ignore the potential of their resources to exhibit such abrupt changes towards irreversible low productive states. Using a combination of statistical changepoint analysis and stochastic cusp modelling, here we show that Western Baltic cod is beyond such a tipping point caused by unsustainable exploitation levels that failed to account for changing environmental conditions. Furthermore, climate change stabilizes a novel and likely irreversible low productivity state of this fish stock that is not adapted to a fast warming environment. We hence argue that ignorance of non-linear resource dynamics has caused the demise of an economically and culturally important social-ecological system which calls for better adaptation of fisheries systems to climate change.

## Introduction

The potential existence of tipping points in dynamic systems is an active field of research because they imply unexpected and sudden changes that are difficult or even impossible to reverse^1,2^. Understanding processes leading to tipping points is also of crucial importance for fisheries management since ignoring such non-linear and discontinuous dynamics can cause unexpected collapses of ecologically, culturally and economically important resources^3^, and may render subsequent recovery efforts unsuccessful^4^. However, present-day fisheries management systems still largely ignore the potential of their resources to exhibit such abrupt changes towards irreversible low productive states^5,6^. Here we provide multiple lines of evidence to show that the ongoing demise of the fisheries on Western Baltic cod (*Gadus morhua*) occurs because this fish stock is beyond a tipping point caused by unsustainable exploitation levels.

**Figure 1:**
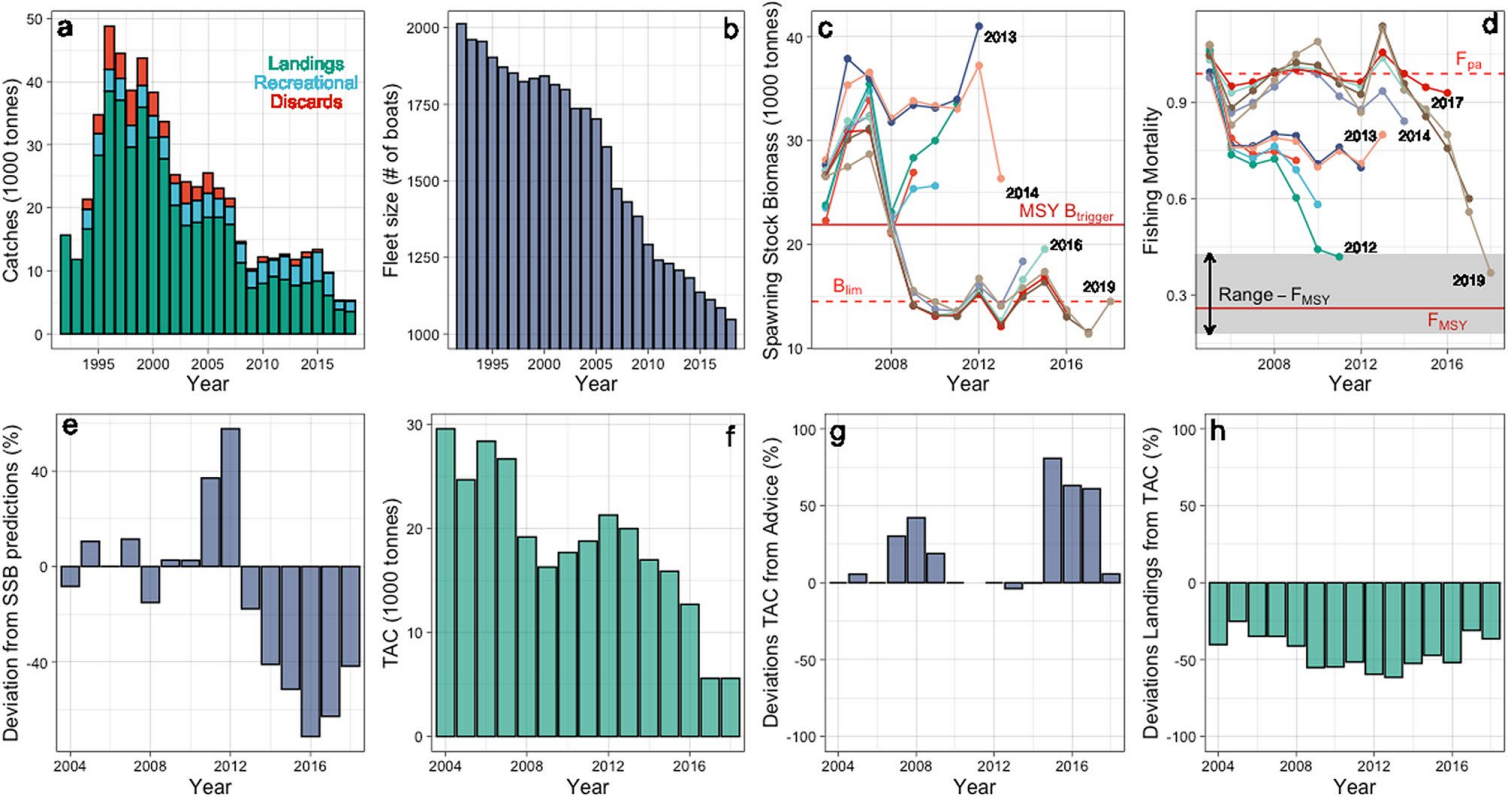
The demise of the Western Baltic cod fishery and challenges to the governance system. (**a**) Catches divided into commercial and recreational fisheries landings as well as discards^13^. (**b**) Size of the German coastal gillnetter fleet. (**c**) Comparison of SSB from stock assessments in years 2008–2019; years indicate assessment years; red solid horizontal line indicates present biomass reference level MSY B_trigger_; red dashed horizontal line indicates biomass reference level B_lim_ indicating impaired reproductive success. (**d**) Comparison of fishing mortality estimates (F) from stock assessments in years 2008–2019; years indicate assessment years; red solid horizontal line indicates present F management target F_MSY_; grey shaded area represents F management range; red dashed horizontal line indicates former precautionary F reference level F_pa_. (**e**) Deviations of realized spawning stock biomass (SSB) from predictions two years before. (**f**) Total allowable catches (TAC) agreed by the EU council of minister. (**g**) Governance system performance evaluated by comparing TAC and advice. (**h**) Governance system performance evaluated by comparing TAC and realized landings by the fishery. Data in (**a**), (**f**), (**g**) and (**h**) from^13^; data in c derived from the German Federal Office of Agriculture and Food (BLE), www.ble.de; for data in (**c**), (**d**), and (**e**) see Supplementary Methods S1.

The social-ecological fisheries system (SEFS) of the German Western Baltic Sea consists of a fishing fleet of a few fresh-fish cutter trawlers (< 24 m in length) but is dominated by c. 1100 small boats (4–10 m in length) that operate gillnets within sight of the coast^7^. This small-scale fishing fleet is responsible for only 4% of the entire German catches, but has a considerable socio-cultural value for local coastal communities as well as socio-economic importance as it supports local employment and attracts tourism to the area^7,8^. This SEFS is presently at the brink of a collapse. Catches of the main resource species cod (*Gadus morhua*) decreased dramatically to less than 10% of those during the late 1990s (Fig. 1a), causing overall landed value to diminish (Supplementary Fig. S1). Consequently, a dramatic and ongoing demise of the fleet is observed with a 50% reduction of the number of fishing boats (Fig. 1b). A further symptom for the ongoing transformation of the SEFS is an increased importance of the recreational fishing sector which nowadays contributes up to > 50% of the entire catches (Supplementary Fig. S2), posing additional threat to traditional fishing livelihoods.

An important obstacle towards halting the unsustainable development of the Western Baltic cod SEFS is large uncertainty in the assessment of the state and dynamics of the fish stock and a poor performance of the fisheries governance system. For example, updated input data for the stock assessment after 2014 changed estimates of spawning stock biomass (SSB) from being above the critical biomass level (MSY B_trigger_; Supplementary Table S1) to a size where reproductive success is impaired (B_lim_) (Fig. 1c). Equivalently, the perception of exploitation pressure changed from fishing mortalities (F) being close to the present management target (F_MSY_; F leading to maximum sustainable yield) to be almost at the level where the stock is considered endangered (F_lim_) (Fig. 1d). Only recently, EU management was able to reduce F into the target range given by the multiannual management plan^9^. Further uncertainty lies in the ability to anticipate future stock trajectories needed for setting total allowable catches (TAC). Since 2013, predictions of SSB were partly more than 60% higher of what was subsequently observed (Fig. 1e). Eventually, the EU fisheries management system failed to halt the demise of the stock by setting TACs (Fig. 1f) constantly higher than scientifically advised (Fig. 1g). Moreover realized landings were generally far below the TAC (Fig. 1h) indicating too high, unbinding quotas and hence a largely unregulated fishery.

We can hence conclude that we find the Western Baltic cod SEFS in a state of collapse and fisheries management failed to protect this now endangered resource species important for the sustainable development of the German Baltic coastal community. We here argue that these failures in the governance system are symptoms of a tipping point realized in Western Baltic cod stock dynamics. Tipping points are related to the concept of regime shifts characterized by abrupt changes in temporal developments as well as structural changes in the internal functioning of a system that are difficult or even impossible to revert^10–12^. We here provide evidence that during the early 2000s Western Baltic cod has realized a tipping point to a low productive state that was caused by recruitment overfishing and is stabilized by ongoing climate change.

## Results

### Abrupt changes and regimes in Western Baltic cod

**Figure 2:**
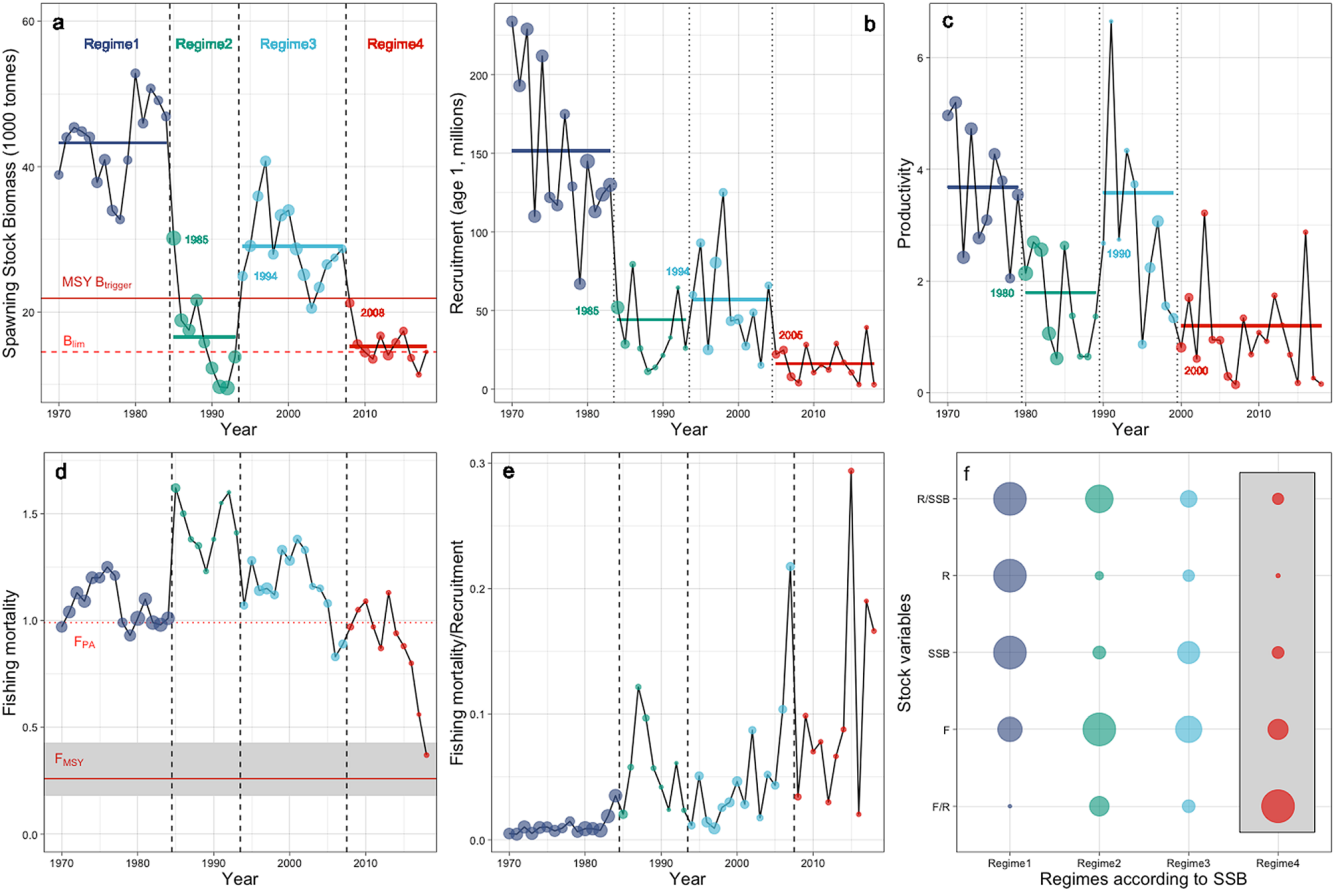
Abrupt changes and regimes in Western Baltic cod. (**a**) Spawning Stock Biomass (SSB); red solid horizontal line represent the biomass reference level MSY B_trigger_; red dashed horizontal lines indicates biomass reference level B_lim_ indicating danger of collapse; points size scaled to fishing mortality. (**b**) Recruitment (R) to the stock, i.e. year-class strength at age 1; point size scaled to SSB. (**c**) Productivity of the stock, i.e. R/SSB; point size scaled to SSB. (**d**) Fishing mortality (F); red solid horizontal lines indicate present F management target F_MSY_; grey shaded area represents F management range; red dashed horizontal line indicates former precautionary F reference level F_pa_; point size scaled to SSB (**e**) Fishing mortality relative to recruitment (F/R); point size scaled to SSB. (**f**) Summary of regime dynamics, point size represents mean of SSB regime periods (see Fig. 1a) with the highest value of a variable scaled to 1; grey shaded area represents recent Regime 4 (see Fig. 1a). Years in a-c indicate major changepoint years. Vertical and dashed lines indicate SSB regimes; vertical dotted lines indicate R regimes in b and R/SSB regimes in c.

A first order indicator for regime shifts in a dynamic system such as an exploited fish stock is the occurrence of abrupt changes^10,14–16^. We tested for abrupt changes in time series of Western Baltic cod spawning stock biomass (i.e., SSB), recruitment (R; the number of age 1 individuals entering the fishable stock or initial year-class strength) and R/SSB (an index for the productivity of the stock) using statistical change point analysis (see “Methods”). The analysis indicated abrupt changes in all three variables separating four distinct regimes (Supplementary Table S2). Intermediate regime 2 and the recent regime 4 show low values of SSB, R and R/SSB (Fig. 2a–c), indicating stock collapses because stock sizes (i.e., SSB) are close or even below B_lim_, the level at which reproductive capacity is impaired (Supplementary Table S1). Our analyses furthermore revealed that abrupt changes in stock productivity (R/SSB) always occurred earlier than those of SSB and R, which largely changed in parallel (only the shift in R to regime 4 preceded the change in SSB). Overall, we find the historical development of Western Baltic cod to be characterized by multiple abrupt changes indicating regime shift dynamics. More importantly, we see that the fish stock is likely nowadays locked in a historically unproductive state that has its roots in the early 2000s.

We subsequently analysed the role unsustainable fishing levels potentially played in the demise of the Western Baltic cod stock, and related regimes suggested by changepoint analysis to respective levels of fishing pressure (Fig. 2d–f). Annual fishing mortalities (F) were generally excessively high (mainly > 1.0, especially during regime 2; Fig. 2d), which translates into annual exploitation rates of > 100% of SSB (indicating additional fishing pressure on immature parts of the population). A simple visual analysis shows that variability in F can explain the dynamics of SSB for the first three regimes, but not for the present low productivity regime (Fig. 2d). Recent regime 4 is characterized by Fs similar to regime 1, while the SSB level is way lower. Even record low F values in recent years (2016–2018) did not lead to an increase in SSB.

The lack of Western Baltic cod recovery shows an apparent disconnect between the pressure regulated by management (F) and the stock size variable (SSB). In regime shift theory such a change in a pressure-state relationship is assumed to be due to a third influential variable gaining importance^10,15^. We here hypothesize that the prevailing unusual low productivity of the stock is responsible for the apparently vanishing importance of F. We hence normalised the original fishing pressure measure (F) by annual year-class strength (R), demonstrating that when accounting for the decrease in the annual production of incoming individuals, fishing pressure is effectively not declining (Fig. 2e). Rather, F/R better explains overall SSB dynamics and especially demonstrates that fishing pressure during the recent regime is on average record high when considering the existing low productivity state. Altogether, our changepoint analysis and the visual inspection of regimes revealed that the recent demise of the cod stock is due to unprecedented low productivity and year-class strength of the stock in parallel to the highest fishing pressure indicated by F/R (Fig. 2f).

### Breakpoints in Western Baltic cod stock functioning

**Figure 3:**
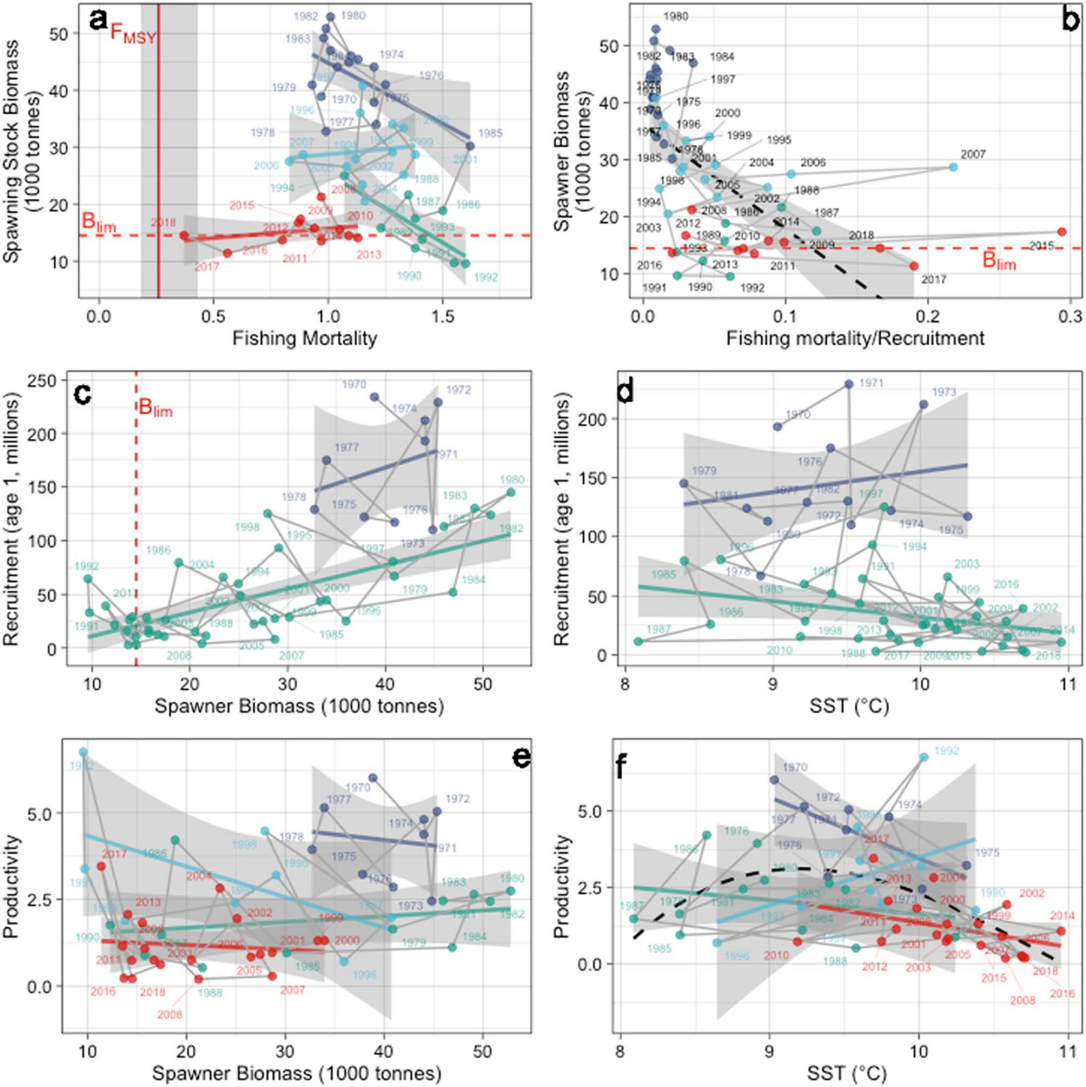
Breakpoints in Western Baltic cod stock functioning. (**a**) Apparent hysteresis in the relationship between spawning stock biomass (SSB) and fishing mortality (F); red vertical and horizontal lines indicate biomass reference level B_lim_ and fishing mortality (F) reference point F_MSY_; vertical grey shaded area represents F management range. (**b**) Effect of scaled exploitation pressure, i.e. fishing mortality relative to recruitment (R, i.e. year-class strength at age 1) on SSB; horizontal line indicates biomass reference level B_lim_; black dashed line indicates alternative linear model (fitted to the data excluding 2007 and 2015 considered as outliers). (**c**) Effect of SSB on R; red vertical and horizontal lines indicate biomass reference level B_lim_. (**d**) Effect of sea surface temperature (SST) on R. (**e**) Effect of SSB on productivity (R/SSB). (**f**) Effect of SST on productivity; black dashed line indicates alternative spline model. Coloured points and lines indicate regime-dependent linear models from breakpoint analysis.

A second indication for regime shift dynamics to be at play are non-stationary relationships in the functioning of a system^15,17,18^. We hence conducted a breakpoint analysis in important bivariate functional relationships governing the dynamics of the Western Baltic cod stock (Fig. 3). We here made a distinction between changepoint analysis that test for a *change* in a time-series (see above) and breakpoint analysis that tests for a *break* in the relationship between two time-series (see “Methods”) We found significant breakpoints in all six functional relationships we investigated, providing additional evidence for regime shift dynamics to govern Western Baltic cod stock development (Supplementary Table S2).

Breakpoints in the relationship between spawning stock biomass (SSB) and fishing mortality (F) display the same decadal pattern we observed in the changepoint analysis of SSB time-series (Fig. 2a), separating two regimes with high stock levels (1970–1985 and 1994–2007) from two low SSB periods (1986–1993 and 2008–2018) (Fig. 3a). Our analysis revealed that F is negatively related to SSB during the first half of the entire observation period. However, since the mid-1990s and especially in regime 4, we find F to be decoupled from the SSB development supporting our visual inspection of time-series patterns above (Fig. 2). The F-SSB relationship hence apparently indicates a strong hysteresis effect of the Western Baltic cod stock size to the reduction in F, a typical characteristic for systems underlying regime shift dynamics^10,14,18^. However, validating our visual inspection of Western Baltic cod time-series (Fig. 2), a different appreciation of the response of SSB to fishing pressure is achieved when scaling F to year-class strength, i.e. recruitment (F/R) (Fig. 3b). Our breakpoint analysis revealed four periods with variable but largely negative effects of F/R on SSB (Fig. 3b, Supplementary Fig. S3). But, over the entire observation period a clear negative effect of F/R is suggested (indicated by a linear regression line in Fig. 3b). Especially in many years of the recent regime (e.g. 2017 and 2018) exceptionally high F/R fishing pressures were related to unsustainably and dangerously low SSB levels (i.e. below B_lim_). Our analysis hence reassures findings above that the apparent hysteresis in the relationship between SSB and F is the result of a drastically reduced productivity of the stock which becomes evident when scaling the fishing mortality F to year-class strength (i.e. R).

In a second set of breakpoint analyses we investigated the effects of SSB on R (i.e. the classical stock-recruitment relationship in fisheries science^19^, but also the effect of ocean warming through climate change represented by sea surface temperature (SST). We found only one breakpoint in both functional relationships, separating a longer period with lower R since the late 1970s/early 1980s from the years before (Fig. 3c,d). Since this breakpoint, the relationship between R and SSB remained strongly linear lacking the typical density-dependent decline in R at high SSB as suggested by traditional models used in fisheries science (e.g. Ricker and Beverton-Holt models)^19^. Moreover, the SSB-R relationship suggests that especially since c. 2000, low R is associated to stock sizes too low (SSB partly below B_lim_) to produce larger year-classes which would be needed for the recovery of the stock (Fig. 3c). Contrary to SSB, we found a weakly positive effect of SST on R before the breakpoint, while afterwards constant warming seemed to have an increasingly negative effect on year-class strength (Fig. 3d). Together both relationships unveil how record low SSB and highest temperatures (recorded at the end of the assessment period) are related to the recently low R regime, suggesting a cumulative effect of fishing and climate change to be responsible for the present lock-in of the stock in an unsustainable state.

Eventually, we investigated breakpoints in the functional relationships between R/SSB (as an index of stock productivity) and both SSB and SST (Fig. 3e,f). We again found three breakpoints separating four regimes in the functioning of the Western Baltic cod stock. Over the entire observation period productivity (R/SSB) showed a weak and variable relationship to SSB and remained recently generally low at low stock sizes (Fig. 3e). Hence, there seems to be generally no overall density-dependent compensation in this cod population, i.e. no increase in R/SSB in response to declining SSB, that would be necessary for a recovery of the stock (Fig. 3e). Such a lack of a compensatory response is especially visible during the last period (since the late 1990s) where low R/SSB is associated with low stock biomass (i.e. SSB). In parallel, we observed that overall SST has a bell-shaped effect on productivity (again with the exception of the intermediate higher SSB period), but a linear negative relationship since the late 1990s where lowest productivity is associated with the highest temperatures during the assessment period (Fig. 3f). Our analyses of functional relationships of R/SSB hence revealed that the recent increase in temperatures likely strongly decreased the productivity of the stock preventing a compensatory response to low SSB that would be needed for a recovery of the Western Baltic cod stock.

In toto, our analysis of breakpoints in functional relationships provided further evidence for regime shift dynamics in the Western Baltic cod stock. Moreover, our analysis indicates that a decrease in productivity since the late 1990s is likely initiated by a reduction in SSB due to unsustainable fishing pressures during almost the entire observation period (with generally F > 1.0). Nowadays, fishing pressure is still too high for the existing reproductive potential and additionally warming of the Western Baltic Sea in response to climate change contributes to a lock-in of the stock in a low productivity regime.

### Hysteresis and stable states in Western Baltic cod

**Figure 4:**
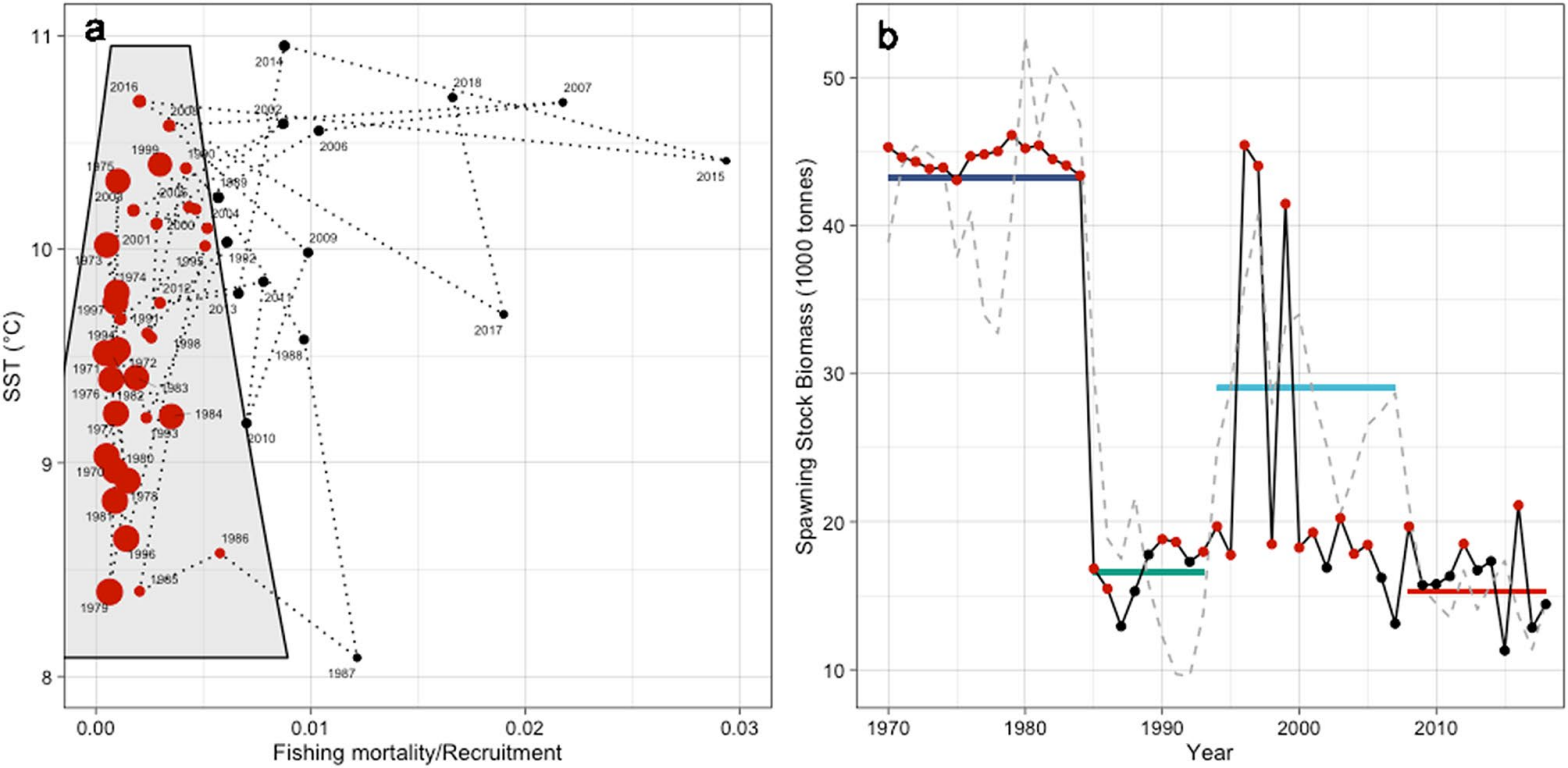
Hysteresis and stable states in Western Baltic cod. (**a**) Effect of scaled exploitation pressure, i.e. fishing mortality (F) relative to recruitment (R, i.e. year-class strength at age 1), and sea surface temperature (SST) on spawning stock biomass (SSB; points scaled to predictions from stochastic cusp modelling); grey shaded polygon indicates the cusp area where bistability of the system exists. (**b**) Time-series on observed (grey dashed line) and predicted SSB (points and black solid line) from stochastic cusp modelling; horizontal coloured bars indicate SSB regimes (see Fig. 1). In (**a**) and (**b**) red points indicate the system to be in the bistable cusp area (see polygon in **a**) and black points indicate a stable system, outside the cusp area.

The ultimate evidence for regime shifts in a dynamic system is the demonstration of the existence of alternative stable states, a goal which is generally difficult to achieve with empirical data only^10,14,15,18^. An approach to evaluate stability patterns from empirical data is stochastic cusp modelling (SCM)^20^. SCM is based on catastrophe theory, popular in the 1970s^21,22^, but recently rediscovered in a number of research fields^23–31^ including fisheries science^4,32^. The *cusp* is one of seven geometric elements in catastrophe theory and represents a 3D surface combining linear and non-linear responses of a state variable to one control variable (called the *asymmetry* variable) modulated by a second so-called *bifurcation* variable^21^ (see “Methods” and Supplementary Fig. S4). In SCM the cusp is represented by a potential function that can be fit to data using the method of moments and maximum likelihood estimators, and the state, asymmetry and bifurcation are canonical variables fit themselves using linear models of observed quantities^20^. Importantly, using SCM we can identify hysteresis by distinguishing between unstable (in fact bistable) and stable states in the dynamics of the cod stock using a statistic called *Cardan´s discriminant* (see “Methods”). Bistable dynamics exist in the non-linear part of the *cusp* under the folded curve, where the state variable can flip between the upper and lower shield, also called the *cusp area* (shaded in light blue in the 3D—Supplementary Fig. S4—and 2D representations of the model surface; Fig. 4a). Outside the *cusp area* the system is assumed to be stable which indicates a high degree of irreversibility (i.e. hysteresis). As suggested in the SCM literature, we conducted a comprehensive model validation that revealed our fitted SCM to be superior to alternative linear and logistic models, explaining a large portion of the variability in the data and fulfilling additional criteria for this model type to be valid^20^ (see “Methods” and Supplementary Table S4).

In our analysis of Western Baltic cod we modelled the dynamics of the state variable as a function of spawning stock biomass (SSB), and the asymmetry and bifurcation variables were fit to time-series of our updated measure of fishing pressure F/R (that adjusts the fishing mortality F to recruitment R; see above) and SST, respectively. Our model setup is based on the results of the previous change- and breakpoint analyses that indicated the importance of F/R and ocean warming of the Western Baltic Sea (represented by SST) for local cod regime dynamics. The model setup hence bears the assumption that SST can alter the relationship between F/R and SSB from linear to non-linear and vice versa (Supplementary Fig. S4).

Projecting the 3D SCM surface on a 2D plain (Supplementary Fig. S1) demonstrates how the interaction between our updated measure of fishing pressure (F/R) and warming (indicated by SST) caused a lock-in of Western Baltic cod in a low and unsustainable SSB state (Fig. 4). Our fitted SCM suggests that warming alters the relationship between SSB and F/R from non-linear discontinuous (at the lower left part of the state space) towards linear and continuous (towards the upper right part of the state space) (Fig. 4a). The first collapse of SSB in the mid 1980s and the intermediate recovery during the mid 1990s occurred when cod stock dynamics can be assumed unstable, i.e. the state variable resided in the bistable cusp area (grey shaded area in Fig. 4a and indicated by red dots in Fig. 4b). Similarly, the stock collapsed again during the late 1990s, but now moving progressively into a state of increasing stability (i.e. the distance of the black dots from the cusp area is increasing in Fig. 4a). The stability of the recent collapsed and depleted state is hence caused by excessive fishing pressure for the level of year-class strength the stock is able to produce (i.e. F/R) and increasing SST in the Western Baltic. Unfortunately, the increased stability in the dynamics of the Western Baltic cod stock implies that a recovery of the resource is presently very unlikely or will at least be very slow. Given that global climate change^33^, and warming of the Baltic Sea will continue^34^, a recovery of the cod stock would need low fishing pressures over a long time allowing for a slow rebuilding of SSB and hence increasing likelihoods of high R supporting a recovery^4^.

## Discussion

Our study demonstrated that Western Baltic cod is beyond a tipping point. Tipping points are related to the concept of regime shifts^10–12^ and our study adds to the growing evidence for regime shifts to be important and increasingly prevalent phenomena in marine ecosystems^12,14,35–39^ and marine fish populations^4,40–42^. We here provided mulitple lines of evidence for such non-linear and non-stationary dynamics by addressing three indicators of regime shifts, i.e. (i) abrupt changes in time-series of population variables, (ii) changing functional relationships among these variables and between external drivers, and (iii) evidence of a recently developed alternative and unfavourable stable state due to the interaction of unsustainable fishing pressure and climate change. We hence provide an approach that allows for detecting these specific characteristics of regime shift dynamics which can serve as a general template for analyzing the existence of these phenomena in many other ecological systems based on empirical data. Change- and breakpoint analyses are well established techniques, but here we especially introduced the potential of the stochastic cusp model (SCM) to reveal stability patterns of regimes. While used in a diversity of scientific fields, SCM is only recently applied to ecological questions and likely needs development with respect to accounting for autocorrelation in time series and known uncertainties in model comparison using indices such as R^2^ and AIC^20,28,43^. We hence followed a careful approach to validate our modelling results using multiple criteria as suggested and applied in other studies^4,32^. We are however convinced that the application of the SCM allowed us to demonstrate how the interactive effects of unsustainable fishing pressure and ocean warming caused the lock-in of the Western Baltic cod stock in a low productivity state, a potential that could be useful for further applications to populations and communities governed by similar dynamics. Our approach can potentially be complemented with other novel methodology to unveil non-linear and state-based dynamics^44^.

We found here that unsustainable exploitation levels reduced the biomass of the adult mature population of Western Baltic cod to levels that impair reproductive success, i.e. the production of larger year-classes that would be able to initiate a recovery of the stock. Low or even declining productivity at low populations sizes suggests depensatory processes to inhibit recovery of the cod stock^45^. The prevalence of depensation in exploited fish stocks, also referred to as the Allee effect, is a long-standing discussion in fisheries science^46^. Empirical evidence for Allee effects is generally lower than for compensatory effects, i.e. increasing productivity at low stock sizes and hence high recovery potential^46^. However, indications for depensation have especially been shown for cod populations^47–51^, but also other species^52–54^. Potential mechanisms of the Allee effect in marine fish populations include limited mating success^48^ and lowered egg fertilization rates^55^, reduced antipredator vigilance^56^, decreased genetic variation among offspring^57^, and predation effects on adults^51,58–60^ as well as early-life history stages^47,61^. Processes causing depensation in Western Baltic cod are however largely unknown (but see Ref.^62^), since knowledge on recruitment processes in Western Baltic cod is generally deficient^63,72^. Our study furthermore revealed that warming of the Western Baltic Sea is related to low cod stock biomass and productivity, and that temperature interacted with overfishing to create a stable unproductive state. Hence, our results support recent findings that interactions between fishing and climate change can increase the impact of the Allee effect, exacerbating the risk of population collapse and causing recovery failure^64^.

Our analysis is based on typical fisheries data that are output of stock assessment models. These data carry inherent uncertainties (see also Fig. 1e) and the variables are not independent of each other, and their use and analysis have well-known shortcomings^46^. Nevertheless, it has been widely acknowledged that rigorously analyzing this type of data can lead to new and important insights in the dynamics of fish stocks as shown here^4,32,65,66^. Future analyses of non-linear dynamics of fish should ideally be based on data sampled in the field, but these are presently not available for long enough periods to test for regime shift dynamics (as is the case for Western Baltic cod). Generally, knowledge on the effects of climate change on Western Baltic cod is lacking. Recent studies suggest warming of shallow-water areas causing reduced food intake and subsequent growth^67^. Other candidate processes are effects on transport and survival of early-life stages^68–70^. Furthermore, experimental studies in combination with ecological-economic modelling have demonstrated that in the future warming and acidification will likely have a negative effect on recruitment with consequences for stock dynamics and harvesting potential^71,72^. However, there is a large knowledge gap on the ecology and population dynamics, and especially recruitment of Western Baltic cod in relation to warming that is expected to continue in the future also in the Western Baltic Sea^34^.

We found ignorance of productivity changes in the management of the stock, both in scientific advice and subsequent quota setting to be a major reason for the collapse of Western Baltic cod. Accounting for productivity changes (here year-class strength) is a first principle of ecosystem-based fisheries management (EBFM) and modelling studies have demonstrated that considering environmental changes can prevent fish stock collapses and can help accounting for the effects of climate change^72–75^. EBFM implementation is lacking behind in the EU compared to the United States^76–80^, while practical use of environmental information in fisheries management is deficient worldwide^81,82^. For example, in the advice framework for EU fish stocks, environmental context, although provided in ecosystem overviews, is still not directly incorporated in the advice. Structured indicator approaches would need to be applied to rigorously assess environmental changes^83–85^. Furthermore, environmental variables need to be included in short-term predictions of fish stocks leading to more realistic evaluations of harvest potentials^86–90^. Those steps towards EBFM would likely alleviate the uncertainties in the appreciation of stock status as shown here for Western Baltic cod. But further steps are needed for implementing a comprehensive EBFM including the use of strategic modelling for evaluating suitable management strategies under climate change^75,91–93^, extended in space^94^ and towards considering multiple species^95,96^. More attention must also be paid to modern monitoring schemes^97^, and especially to the consideration of the human dimension^98,99^ towards a social-ecological systems approach^100^.

Eventually our study revealed that the Western Baltic cod stock is in an unsustainable state. Given the presently low spawning stock size, the continuing warming of the Baltic Sea and the fishing pressure being to high for the productivity of the stock, the prospects for a recovery are low. Unfortunately similar developments presently occur in Western Baltic herring^101^. Consequently, the local social-ecological fisheries system (SEFS) is endangered since the necessity to set low total allowable catches (TACs) by EU fisheries management to protect the resources do not provide sufficient income to support the present fleet size. Overall this mainly small-scale fishery has only a comparatively low direct economic importance at the German Western Baltic coast^7^. But, indirect economic value through its importance for local employment and tourism is assumed to be huge, although not rigorously assessed yet. Socio-cultural importance of the resource and the fisheries is presently demonstrated by a strong media attention that documents the demise of the SEFS in television, radio and newspaper features (Supplementary Table S3). To halt this development and to guide the Western Baltic SEFS into a sustainable future that is resilient to the expected climate change, rigorous climate adaptation planning is needed^102–105^. Adaptation efforts need to include the exploration of the vulnerability of the fish community to climate change^106–109^, but especially an evaluation of potential adaptive measures for the fisheries including diversification of target species, sustainable value chains and livelihood diversification^110,111^. Such information is crucially needed to implement measures that make this fishery climate-ready and sustainable, and to prevent its further collapse which would mean a significant loss of cultural identity for the German Western Baltic coast.

## Methods

### Data

We derived data on Western Baltic cod (*Gadus morhua*) spawning stock biomass (SSB), recruitment at age 1 (R) and fishing mortality (F) reported from model-based stock assessments conducted by the International Council for the Exploration of the Sea (ICES) Baltic Fisheries Assessment Working Group (WGBFAS; www.ices.dk/community/groups/Pages/WGBFAS.aspx). Stock assessment models reconstruct historical population dynamics based on catch data from commercial fisheries supplemented by scientific survey data. Stock assessments for the period 1985–2018 were conducted using a State Space Assessment model (SAM)^112^. We extended the time-series backwards until 1970 using an earlier assessment^113^. We combined these two data sources to be able to analyse a longer period with likely more contrast in regime dynamics. Combining the data sources likely introduced a bias since data from the period before 1985 did not account for mixing with the neighbouring Eastern Baltic cod stock^114,115^. However, since strong mixing is likely a more recent phenomenon we do not consider this bias to severely influence our results.

We used data on sea surface temperature (SST) from the NOAA Extended Reconstructed Sea Surface Temperature dataset (ERSST, www.ncdc.noaa.gov) v.4. and computed mean annual SST values over the management area of Western Baltic cod (i.e. ICES Sub-divisions 22–24).

We furthermore demonstrated the demise of the Western Baltic cod fishery and challenges to the governance system (Fig. 1) using a number of data sources given in the caption of Fig. 1 and Supplementary Methods S1.

### Software

All analyses were conducted using the free software environment for statistical computing and graphics R^116^ (version 3.6.1) within the RStudio environment (version 1.2.5019) using packages *tidyverse*^117^ for data handling and graphics, *changepoint*^118^ for changepoint analysis, *strucchange*^119^ for breakpoint analysis and *cusp*^20^ for stochastic cusp modelling.

### Changepoint and breakpoint analysis

In our study we considered abrupt changes in time-series and changing functional relationships as two key characteristics of regime shift dynamics^15,17,18^. We hence made a distinction between *changepoints* within a single time-series and *breakpoints* in the functional relationship between two time-series. For the former we used the R-package “Changepoint”^117^, while for the latter we used the R-package “strucchange”^119^, a choice based on a comparison of statistical changepoint analyses^120^. In *changepoint* detection methods points are identified at which statistical properties of a sequence of observations change, a problem important in many different scientific fields such as ecology, climatology, bioinformatics, finance, oceanography and medical imaging. We tested for multiple changepoints in the *means* of time-series of Western Baltic cod spawning stock biomass (SSB), recruitment at age 1 (R) and productivity (R/SSB) applying the *cpt.mean* function (R package “Changepoint”). Within the function we used the *Pruned Exact Linear Time* (PELT) algorithm which minimizes a cost function using a maximum likelihood approach to find the optimal number and locations of $m$ changepoints that will split the time-serie into $m+1$ segments. We furthermore used the “Normal” option for the test statistic, the “AIC” as a penalty, and a minimum segment length of 10 years to allow for a reasonable regime length. For the analysis of *breakpoints* in Western Baltic cod functional relationships, we used the *breakpoints* function (R package “strucchange”). The algorithm detects breakpoints in linear regression models by identifying where the coefficients shift between multiple stable regression relationships. The function estimates breakpoints by finding the optimal model with $m$ breakpoints and $m+1$ segments that minimizes residual sum of squares (RSS). We investigated functional relationships representing the effects of fishing mortality (F) and F accounting for R (F/R) on SSB, the effects of SSB and sea surface temperature (SST) on R, as well as the effects of SSB and SST on productivity (R/SSB).

### Stochastic cusp model

We used the stochastic cusp model (SCM) to investigate the stability in Western Baltic cod regimes. SCM is based on catastrophe theory and describes abrupt changes of a state variable (${z}_{t}$) as a result of the interaction between an *asymmetry* $\alpha$ and a *bifurcation* $\beta$ variable. The canonical form of the potential function $V({z}_{t},\alpha ,\beta )$ for the cusp catastrophe is given by:

1 $$-V\left({z}_{t},\alpha ,\beta \right)=-\frac{1}{4}{z}_{t}^{4}+\frac{1}{2}{\beta }_{t}^{2}+\alpha {z}_{t}.$$

Equilibrium points in Eq. (1) as a function of ($\alpha$) and ($\beta$), are solutions to:

2 $$\alpha +\beta {z}_{t}-{z}_{t}^{3}=0.$$

Equation (2) has one solution if the so-called *Cardan’s discriminant* $\delta =27\alpha -4{\beta }^{3}$ is $>1$ and three solutions if $\delta <0$. Projecting the 3D cusp catastrophe on a 2D plain, the set of values of $\alpha$ and $\beta$ for which $\delta =0$ delineates the *bifurcation area* (grey area in Fig. 4a and see Supplementary SI-3). Inside the bifurcation area the system is unstable and stable outside.

Fitting the cusp catastrophe to data is possible through the stochastic differential equation (representing the SCM):

3 $$d{z}_{t}=\left(-{z}_{t}^{3}+\beta {z}_{t}+\alpha \right)dt+{\sigma }_{z}d{W}_{t},$$

where the first part of Eq. (3) is a drift term, ${\sigma }_{z}$ is a diffusion parameter and ${W}_{t}$ represents a Wiener process.

Parameters $\alpha$ and $\beta$ as well as the state variable (${z}_{t}$) can be modelled as linear functions of one or more exogenous variables using a likelihood approach. For our case of Western Baltic cod, we modelled ${z}_{t}$ as a function of cod spawning stock biomass (SSB), $\alpha$ as a function of fishing pressure (F/R) defined as the fishing mortality (F) accounting for year-class strength, i.e. recruitment (R), and $\beta$ as a function sea surface temperature (SST):

4a $${z}_{t}={\omega }_{0}+{\omega }_{1}SSB,$$

4b $${\alpha }_{t}={\alpha }_{0}+{\alpha }_{1}F/R,$$

4c $${\beta }_{t}={\beta }_{0}+{\beta }_{1}SST,$$

where ${\alpha }_{0}$, ${\beta }_{0}$ and ${\omega }_{0}$ are intercepts and ${\alpha }_{1}$, ${\beta }_{1}$ and ${\omega }_{1}$ the slopes of the linear models.

We validated the fitted SCM by assessing (i) the significance of SSB in the linear model of ${z}_{t}$, (i) evidence for the existence of bimodality in ${z}_{t}$ in the cusp area, (iii) the percentage of observations in the cusp area, and (iv) the goodness of the SCM fit using Cobb’s pseudo-R^2^. Moreover, we compared the fitted SCM to alternative linear and logistic regression models often used to confront linear and continuous dynamics with the non-linear discontinuous regime shift case. For results of the model validation see Supplementary Table S4.

## Supplementary Information

Supplementary Information.

## Data Availability

The data that support the findings of this analysis are available upon request. Data sources are provided in the text.
