# Supplementary material for: Tipping point realized in cod fishery
Source: Sci Rep. 2021 Jul 12;11:14259. doi: 10.1038/s41598-021-93843-z (PMC8275682; doi:10.1038/s41598-021-93843-z)
Supplement: Supplementary file 1 — Supplementary Information. [file 41598_2021_93843_MOESM1_ESM.pdf]

Tipping point realized in cod fishery - Supplementary material

Christian Möllmann[1], Xochitl Cormon[1], Steffen Funk[1], Saskia Otto[1], Jörn Schmidt[2,3], Heike Schwermer[1,2], Camilla Sguotti[1], Rudi Voss[2,4] & Martin Quaas[4]

[1]Institute of Marine Ecosystem and Fisheries Science (IMF), Center for Earth System Research and Sustainability (CEN), University of Hamburg, Germany. [2]Department of Economics, Christian-Albrechts-University Kiel, Germany. [3] International Council for the Exploration of the Sea (ICES), Copenhagen, Denmark. [4]Biodiversity Economics, German Centre for Integrative Biodiversity Research (iDiv), Halle-Jena-Leizig, Germany.

Supplementary Material contains:

Supplementary Methods
Methods S1: Evaluation of uncertainty in the perception of the fish stock.

Supplementary Tables
Table S1. Reference points considerd in the management of Western Baltic cod. Table S2. Change- and breakpoint years in Western Baltic cod time-series and functional relationships. Table S3. Examples of recent media attention on the demise of the Western Baltic fishery in German regional and national media. Table S4. Summary statistics of fitted stochastic cusp model (SCM).

Supplementary Figures
Figure S1: Size of the German coastal gillnetter fleet. Figure S2: Proportion of recreational landings. Figure S3: Breakpoint model for the effect of fishing mortality (F) scaled to recruitment (R) on spawning stock biomass (SSB). Figure S4: 3D representation of the cusp model.

Supplementary Methods

Supplementary Methods S1: Evaluation of uncertainty in the perception of the fish stock.
We evaluated uncertainty in the perception of the Western Baltic cod stock first by assessing the *retrospective pattern* (Fig. 1e,f), a systematic inconsistency among successive estimates of stock status based on increasing availability or precision of underlying data, well known in fisheries science.[1] To this end, we compiled data on spawning stock biomass (SSB) and fishing mortalities (F) for years > 2004 derived from stock assessments conducted in years 2006-2019.[2–15] Using these stock asssessment outputs we additionally investigated the *accuracy of short of short-term predictions* of SSB that are needed for setting total allowable catches (Fig. 1g). We therefore compared predictions two years ahead of an assessment year with the subsequently estimated SSB at the observed F and computed the percent deviations.

Supplementary Tables

Supplementary Table S1. Reference points considered in the management of Western Baltic cod.[16] *in tonnes

Reference_point	Value	Description
**F_{MSY}**	0.26	The fishing mortality (F) expected to give maximum sustainable yield (MSY) in the long term
**MSY $B_{trigger}$**	21876*	Lower bound to the spawning stock biomass (SSB) when the stock is fished at F_{MSY}
**B_{lim}**	14500*	Biomass limit below which a stock is considered to have reduced reproductive capacity
**B_{pa}**	21876*	A stock status reference point above which the stock is considered to have full reproductive capacity, having accounted for estimation uncertainty
**F_{lim}**	1.45	F which leads SSB to B_{lim}
**F_{pa}**	0.99	F which leads SSB to B_{pa}
**F_{MSY} range**	0.18-0.43	F_{MSY} range according to EU multiannual plan

Supplementary Table S2. Change- and breakpoint years in Western Baltic cod time-series and functional relationships. SSB-spawning stock biomass, R-recruitment, F-fishing mortality, SST-sea surface temperature.

Variable_Function	Change_Break1	Change_Break2	Change_Break3
**SSB**	1984	1994	2007
**R**	1983	1993	2004
**R/SSB**	1979	1989	1999
**SSB vs F**	1985	1994	2007
**SSB vs F/R**	1985	1993	2007
**R vs SSB**	1979		
**R vs SST**	1982		
**R/SSB vs SSB**	1979	1990	1997
**R/SSB vs SST**	1977	1989	1997

Supplementary Table S3. Examples of recent media attention on the demise of the Western Baltic fishery in german regional and national media.

Media_type	Media_name	Title	Translation	http	Date
**Radio**	NDR Info	Neue Fangquoten: Ostseefischerei vor dem Aus?	New fishing quotas: Baltic Sea fishing before the end?	www.ndr.de/nachrichten/info/Neue-Fangquoten-Ostseefischerei-vor-dem-Aus,fangquoten146.html	15 September 2019
**Radio**	NDR Info	EU-Fangquoten: Ostsee-Fischer befürchten Pleiten	EU fishing quotas: Baltic fishermen fear bankruptcy	www.ndr.de/nachrichten/info/EU-Fangquoten-Ostsee-Fischer-befuerchten-Pleiten,fangquoten148.html	14 October 2019
**Weekly Newspaper**	Der Spiegel	Dorsch- und Hering-Quoten für 2020: Ostsee-Fischfang um mehr als die Hälfte reduziert	Cod and herring quotas for 2020: Baltic Sea fisheries reduced by more than half	www.spiegel.de/wissenschaft/mensch/hering-dorsch-ostsee-fischfang-fuer-2020-reduziert-a-1291593.html	15 October 2019
**Weekly Newspaper**	Die Zeit	Weniger Hering, weniger Dorsch: Ostseefischer empört über Fangbegrenzung	Less herring, less cod: Baltic fishermen outraged by catch limits	www.zeit.de/news/2019-10/15/ostsee-fischfang-fuer-2020-reduziert	15 October 2019
**Daily Newspaper**	Süddeutsche Zeitung	Wenig Verständnis für Fischfangquoten und Förderpolitik	Little understanding of fishing quotas and support policy	www.sueddeutsche.de/wirtschaft/fischerei-stralsund-wenig-verstaendnis-fuer-fischfangquoten-und-foerderpolitik-dpa.urn-newsml-dpa-com-20090101-191120-99-808403	20 November 2019
**Radio**	NDR	EU beschließt Hilfen für alle Ostseefischer NDR	EU decides aid for all Baltic Sea fishermen NDR	www.ndr.de/nachrichten/mecklenburg-vorpommern/EU-beschliesst-Hilfen-fuer-alle-Ostseefischer,fischer1088.html	16 December 2019
**Daily Newspaper**	Der Tagesspiegel	Fangquoten für die Ostsee sinken EU arbeitet an Abwrackprämie für Fischkutter	Fishing quotas for the Baltic Sea down EU working on scrapping premiums for fishing boats	www.tagesspiegel.de/wirtschaft/fangquoten-fuer-die-ostsee-sinken-eu-arbeitet-an-abwrackpraemie-fuer-fischkutter/25344104.html	17 December 2019
**Weekly Newspaper**	Der Spiegel	Bedrohte Existenz von Fischern: Dann müsste ich in Privatinsolvenz gehen	Threatened existence of fishermen: Then I would have to go into private insolvency	www.spiegel.de/wirtschaft/fischfang-in-der-ostsee-was-die-neuen-fangquoten-aus-sicht-eines-fischers-bedeuten-a-40c8a6c6-291b-4aba-9c8d-da75b9cb6513	9 January 2020
**Radio**	NDR 1 Radio MV	Gastronomen in MV sorgen sich wegen Fangquoten	Caterers in MV are worried about fishing quotas	www.ndr.de/nachrichten/mecklenburg-vorpommern/Gastronomen-in-MV-sorgen-sich-wegen-Fangquoten,fangquote148.html	18 January 2020
**Radio**	NDR 1 Radio MV	Usedomer Fischer leiden unter sinkenden Fischfangquoten	Usedom fishermen suffer from declining fishing quotas	www.ndr.de/nachrichten/mecklenburg-vorpommern/Usedomer-Fischer-leiden-unter-sinkenden-Fischfangquoten,fischerei214.html	20 February 2020
**TV**	ZDF	Fischerei in Zeiten von Corona	Fishing in times of Corona	www.zdf.de/dokumentation/planet-e/fischerei-in-zeiten-von-corona-102.html	20 May 2020
**Daily Newspaper**	Frankfurter Allgemeine Zeitung	Die letzten Fischer an der Ostsee	The last fishermen of the Baltic Sea	www.faz.net/aktuell/gesellschaft/menschen/das-sind-die-letzten-fischer-an-der-ostsee-16783172.html	28 May 2020
**Radio**	Deutschlandfunk	Fangquoten in der Ostsee: Ausgefischt?	Fishing quotas in the Baltic Sea: fished out?	www.deutschlandfunk.de/fangquoten-in-der-ostsee-ausgefischt.740.de.html?dram:article_id=477838	7 June 2020
**TV**	ARTE	Der letzte Fang? Ostseefischer vor dem Aus	The final catch? Baltic fishermen before the end	www.arte.tv/de/videos/092187-003-A/re-der-letzte-fang/	11 June 2020

Supplementary Table S4. Summary statistics of fitted stochastic cusp model (SCM). Estimate and standard error (stder) for SCM model parameter (see methods); z statistic and associated probability (*significant at 0.01, **significant at 0.001); R^2 and AIC values for SCM and alternative linear and logistic models; % $cusp_{area}$ indicates the percentage of observations in the cusp area (should be >10).

Statistic	Estimate	stder	z	p
**α_0**	$4.02x10^{-1}$	$2.20x10^{-1}$	1.83	0.0677
**α_1**	-1.58x10^2	$4.97x10^{1}$	-3.18	0.0015*
**β_0**	4.94	3.93	1.26	0.2087
**β_1**	-3.76x10^-1	$4.08x10^{-1}$	-0.92	0.3572
**z_0**	-2.88	$2.47x10^{-1}$	-11.68	<0.001**
**z_1**	9.31x10^-5	$7.91x10^{-6}$	11.78	<0.001**
**R^2_{cusp}**	0.70			
**R^2_{linear}**	0.27			
**$R^2_{logistic}$**	0.59			
**AIC_{cusp}**	118			
**AIC_{linear}**	1057			
**$AIC_{logistic}$**	1030			
**% $cusp_{area}$**	69			

Supplementary Figures

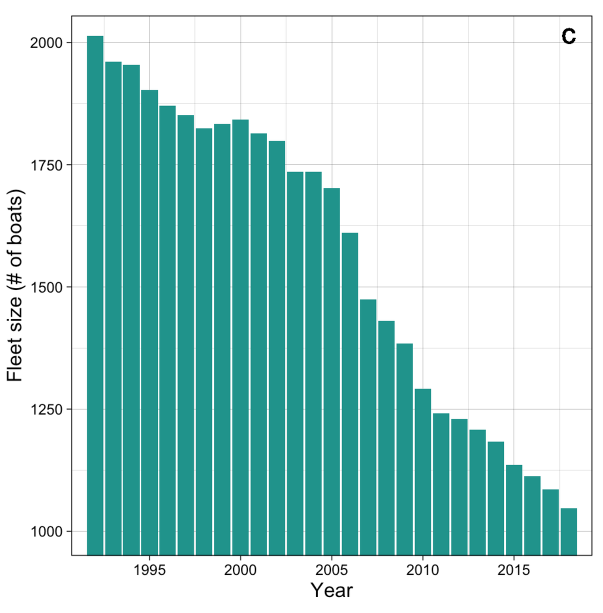

Supplementary Figure S1| Size of the German coastal gillnetter fleet.

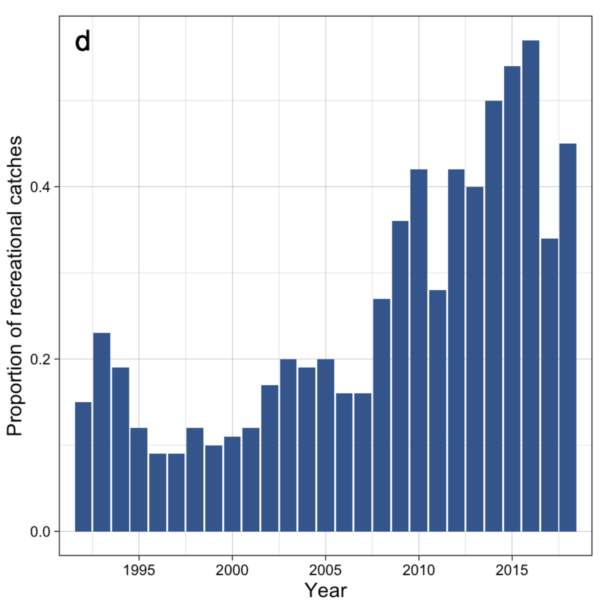

Supplementary Figure S2| Proportion of recreational landings.

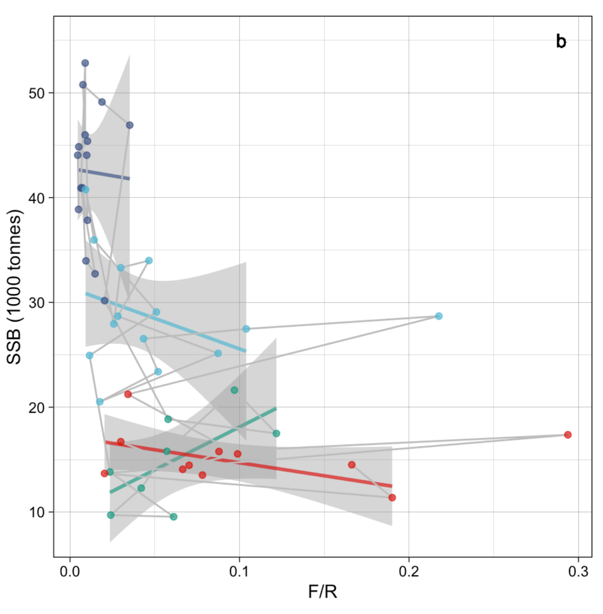

Supplementary Figure S3| Breakpoint model for the effect of fishing mortality (F) scaled to recruiment (R, i.e. year-class strength at age 1) on spawning stock biomass (SSB).

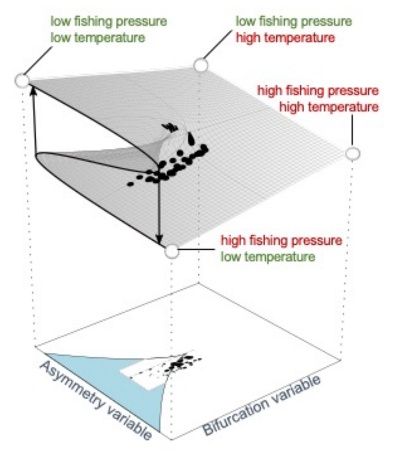

Supplementary Figure S4| 3D representation of the cusp model. The cusp model was fitted with fishing pressure (Fishing mortality/Recruitment - F/R) as a predictor for the *asymmetry variable* and sea surface temperature (SST) as a predictor for the *bifurcation variable*; the 3D surface can be mapped to the 2D *control plain* which represents Fig. 4 and where the *cusp area* (in light blue) that represents the area of multiple equilibria sits below the folded part of the cusp; outside the *cusp area* the system is stable.

Supplementary References

1. Mohn, R. The retrospective problem in sequential population analysis: An investigation using cod fishery and simulated data. *ICES Journal of Marine Science* **56**, 473–488 (1999).

2. ICES. Baltic Fisheries Assessemant Working Group. **ICES CM 2006/ACFM:24**, (2006).

3. ICES. Baltic Fisheries Assessemant Working Group. **ICES CM 2007/ACFM:15**, (2007).

4. ICES. Baltic Fisheries Assessemant Working Group. **ICES CM 2008/ACOM:06**, (2008).

5. ICES. Baltic Fisheries Assessemant Working Group. **ICES CM 2009/ACOM:07**, (2009).

6. ICES. Baltic Fisheries Assessemant Working Group. **ICES CM 2010/ACOM:10**, (2010).

7. ICES. Baltic Fisheries Assessemant Working Group. **ICES CM 2011/ACOM:10**, (2011).

8. ICES. Baltic Fisheries Assessemant Working Group. **ICES CM 2012/ACOM:10**, (2012).

9. ICES. Baltic Fisheries Assessemant Working Group. **ICES CM 2013/ACOM:10**, (2013).

10. ICES. Baltic Fisheries Assessemant Working Group. **ICES CM 2014/ACOM:10**, (2014).

11. ICES. Baltic Fisheries Assessemant Working Group. **ICES CM 2015/ACOM:10**, (2015).

12. ICES. Baltic Fisheries Assessemant Working Group. **ICES CM 2016/ACOM:11**, (2016).

13. ICES. Baltic Fisheries Assessemant Working Group. **ICES CM 2017/ACOM:11**, (2017).

14. ICES. Baltic Fisheries Assessemant Working Group. **ICES CM 2018/ACOM:11**, (2018).

15. ICES. Baltic Fisheries Assessemant Working Group. (2019) doi:10.17895/ICES.PUB.5949.

16. ICES. Benchmark Workshop on Baltic Cod stocks (WKBALTCOD2). (2019) doi:10.17895/ICES.PUB.4984.
